# Early detection of amyloid load using ^18^F-florbetaben PET

**DOI:** 10.1186/s13195-021-00807-6

**Published:** 2021-03-27

**Authors:** Santiago Bullich, Núria Roé-Vellvé, Marta Marquié, Susan M. Landau, Henryk Barthel, Victor L. Villemagne, Ángela Sanabria, Juan Pablo Tartari, Oscar Sotolongo-Grau, Vincent Doré, Norman Koglin, Andre Müller, Audrey Perrotin, Aleksandar Jovalekic, Susan De Santi, Lluís Tárraga, Andrew W. Stephens, Christopher C. Rowe, Osama Sabri, John P. Seibyl, Mercè Boada

**Affiliations:** 1 Life Molecular Imaging GmbH, Tegeler Str. 6-7, 13353 Berlin, Germany; 2 Fundació ACE Institut Català de Neurociències Aplicades, Research Center and Memory Unit - Universitat Internacional de Catalunya (UIC), Barcelona, Spain; 3 Centro de Investigación Biomédica en Red Enfermedades Neurodegenerativas (CIBERNED), Instituto de Salud Carlos III, Madrid, Spain; 4 Helen Wills Neuroscience Institute, University of California, Berkeley and Lawrence Berkeley National Laboratory, Berkeley, CA USA; 5 Department of Nuclear Medicine, University Hospital Leipzig, Leipzig, Germany; 6 Department of Psychiatry, University of Pittsburgh, Pittsburgh, PA USA; 7 Departments of Medicine and Molecular Imaging, University of Melbourne, Austin Health, Melbourne, Victoria Australia; 8 The Australian e-Health Research Centre, Health and Biosecurity, CSIRO, Melbourne, Victoria Australia; 9 Life Molecular Imaging Inc, Boston, MA USA; 10 Invicro, New Haven, CT USA

**Keywords:** Florbetaben, PET, Amyloid-beta, Subjective memory complainers, Mild cognitive impairment, Alzheimer’s disease

## Abstract

**Background:**

A low amount and extent of Aβ deposition at early stages of Alzheimer’s disease (AD) may limit the use of previously developed pathology-proven composite SUVR cutoffs. This study aims to characterize the population with earliest abnormal Aβ accumulation using ^18^F-florbetaben PET. Quantitative thresholds for the early (SUVR_early_) and established (SUVR_estab_) Aβ deposition were developed, and the topography of early Aβ deposition was assessed. Subsequently, Aβ accumulation over time, progression from mild cognitive impairment (MCI) to AD dementia, and tau deposition were assessed in subjects with early and established Aβ deposition.

**Methods:**

The study population consisted of 686 subjects (*n* = 287 (cognitively normal healthy controls), *n* = 166 (subjects with subjective cognitive decline (SCD)), *n* = 129 (subjects with MCI), and *n* = 101 (subjects with AD dementia)). Three categories in the Aβ-deposition continuum were defined based on the developed SUVR cutoffs: Aβ-negative subjects, subjects with early Aβ deposition (“gray zone”), and subjects with established Aβ pathology.

**Results:**

SUVR using the whole cerebellum as the reference region and centiloid (CL) cutoffs for early and established amyloid pathology were 1.10 (13.5 CL) and 1.24 (35.7 CL), respectively. Cingulate cortices and precuneus, frontal, and inferior lateral temporal cortices were the regions showing the initial pathological tracer retention. Subjects in the “gray zone” or with established Aβ pathology accumulated more amyloid over time than Aβ-negative subjects. After a 4-year clinical follow-up, none of the Aβ-negative or the gray zone subjects progressed to AD dementia while 91% of the MCI subjects with established Aβ pathology progressed. Tau deposition was infrequent in those subjects without established Aβ pathology.

**Conclusions:**

This study supports the utility of using two cutoffs for amyloid PET abnormality defining a “gray zone”: a lower cutoff of 13.5 CL indicating emerging Aβ pathology and a higher cutoff of 35.7 CL where amyloid burden levels correspond to established neuropathology findings. These cutoffs define a subset of subjects characterized by pre-AD dementia levels of amyloid burden that precede other biomarkers such as tau deposition or clinical symptoms and accelerated amyloid accumulation. The determination of different amyloid loads, particularly low amyloid levels, is useful in determining who will eventually progress to dementia. Quantitation of amyloid provides a sensitive measure in these low-load cases and may help to identify a group of subjects most likely to benefit from intervention.

**Trial registration:**

Data used in this manuscript belong to clinical trials registered in ClinicalTrials.gov (NCT00928304, NCT00750282, NCT01138111, NCT02854033) and EudraCT (2014-000798-38).

## Background

Extracellular amyloid-beta (Aβ) aggregates are a key pathologic hallmark of Alzheimer’s disease (AD) [1]. Aggregation of Aβ is a slow and protracted process which may extend for more than two decades before the onset of clinical symptoms [2]. The lack of success of anti-Aβ therapeutic clinical trials in reducing the cognitive decline in AD [3, 4] has encouraged investigators to start intervention at the earliest possible phase when abnormalities in amyloid biomarkers are detectable even at the asymptomatic stage [5–8].

Amyloid positron emission tomography (PET) with ^18^F-florbetaben is an established biomarker of Aβ deposition [9]. Visual assessment of ^18^F-florbetaben PET is used in the clinical setting to estimate Aβ neuritic plaque density and to classify scans as Aβ-positive or Aβ-negative. Visual assessment was validated against histopathological confirmation of the presence of Aβ deposition [9, 10], but it is dichotomous and may lack sensitivity to assess longitudinal changes. In the research setting, a quantitative approach using composite standardized uptake value ratios (SUVRs) calculated from selected cerebral cortical areas is currently being used as a screening tool in clinical trials and is able to detect Aβ changes either in clinical trials after an anti-Aβ drug is administered or in longitudinal observational studies [11]. ^18^F-Florbetaben PET SUVR abnormality cutoffs have also been developed to accurately categorize scans [12]. An SUVR abnormality cutoff of 1.478 in a global cortical composite region relative to the cerebellar cortex was developed using histopathological confirmation as the standard of truth providing excellent sensitivity (89.4%) and specificity (92.3%) to detect established Aβ pathology [9]. Other groups have developed other SUVR abnormality cutoffs for ^18^F-florbetaben PET ranging from 1.38 to 1.45 using different populations, analytical methods, and standards of truth [10, 12–18]. These SUVR cutoffs, however, were developed with the aim of discriminating between subjects with established Aβ pathology (e.g., AD) and other populations (e.g., cognitively normal elderly subjects). Therefore, these global SUVR cutoffs are not optimal to detect the earliest abnormal pathophysiological accumulation of amyloid load and do not provide topographical information. In addition, several studies have shown that measures of Aβ deposition below a threshold of established Aβ pathology carry critical information on initial pathological brain changes and may indicate appropriate time periods for interventions [19]. Moreover, the regional evolution of Aβ load may enable earlier identification of subjects in the AD pathologic continuum and may overcome dichotomous measures [20]. Regional information has shown to be relevant in staging Aβ pathology [21–23], tracking disease progression, and assessing the risk of cognitive decline [23–25].

The aim of this study was to characterize the population with the earliest abnormal pathophysiological Aβ accumulation using ^18^F-florbetaben PET and to identify those subjects that will likely accumulate Aβ over time. To this end, a sample of young cognitively normal subjects (20–40 years) scanned with ^18^F-florbetaben PET was used to develop regional SUVR cutoffs to detect early Aβ accumulation. Subsequently, the topography of abnormal Aβ accumulation, Aβ accumulation over time, progression to AD dementia, and tau deposition were assessed in older cognitively impaired or cognitively unimpaired individuals with early and established Aβ accumulation.

## Materials and methods

### Participants

The study population consisted of 686 subjects who underwent at least one ^18^F-florbetaben PET and T1-weighted MRI scans in established research cohort studies summarized in Table 1. The clinical diagnosis of the study participants included young and cognitively normal healthy controls (20–40 years) (yHC, *n* = 65), elderly healthy controls (eHC, *n* = 223), subjects with subjective cognitive decline (SCD, *n* = 168), subjects with mild cognitive impairment (MCI, *n* = 129), and subjects with AD dementia (*n* = 101). The sample of yHC (*n* = 65) (dataset #1) was used to develop an SUVR cutoff for early Aβ accumulation. A sample of eHC (*n* = 66) and AD (*n* = 73) subjects (dataset #2) was used to develop an SUVR cutoff for established Aβ pathology using ROC analysis. A subset of participants with SCD and MCI (*n* = 212) (datasets #3 and #4) underwent two or three ^18^F-florbetaben PET scans to assess Aβ deposition over time. A subset of MCI subjects (datasets #4) that underwent three ^18^F-florbetaben PET scans at baseline (*n* = 44), 1 year (*n* = 40), and 2 years (*n* = 35) and a 4-year clinical follow-up was used to assess conversion to AD dementia in addition to Aβ deposition over time. Another subset of participants (dataset #5) (*n* = 157 (eHC), *n* = 85 (MCI), *n* = 28 (AD)) underwent a ^18^F-flortaucipir PET in addition to the ^18^F-florbetaben PET to assess the association between Aβ and tau deposition. Subjects from the ADNI study were not assessed visually. The demographic characteristics of the samples and image acquisition methods are summarized in Table 1 and supplemental material 1.

**Table 1:** Summary of the participants in the study

Dataset identifier	Source	Clinical diagnosis	Number	Age	M/F	Methods
#1	NCT00928304^†^	yHC	65	27.4 ± 5.1	25/40	Sample of yHC (20–40 yrs) that underwent a ^18^F-florbetaben PET scan. This subset was used to develop an SUVR cutoff for early Aβ accumulation.
#2	NCT00750282 [13]	eHC AD	66 73	68.0 ± 6.9 71.0 ± 7.9	28/38 41/32	All subjects underwent a ^18^F-florbetaben PET scan. This subset was used to develop an SUVR cutoff for established Aβ pathology using ROC analysis.
#3	EudraCT: 2014-000798-38 [26]	SCD	168	64.9 ± 7.3	65/103	SCD patients from the Fundació ACE Healthy Brain Initiative (FACEHBI) study that underwent two ^18^F-florbetaben PET scans at baseline and after 2 years. This subset was used to assess the Aβ accumulation over time.
#4	NCT01138111 [18]	MCI	44	72.6 ± 6.6	28/16	MCI subjects that underwent three ^18^F-florbetaben PET scans at baseline (*n* = 44), 1 yr (*n* = 40), and 2 yrs (*n* = 35) and a 4-year clinical follow-up. This subset was used to assess the Aβ accumulation over time and conversion to AD.
#5	NCT02854033 (ADNI3^‡^)	eHC MCI AD	157 85 28	70.6 ± 6.1 71.7 ± 8.1 71.3 ± 7.0	62/95 47/38 18/10	Subjects from the ADNI3 study that underwent a ^18^F-florbetaben PET and a ^18^F-flortaucipir PET. This subset was used to assess the association between Aβ and tau deposition.

^†^Unpublished methods on the sample of yHC are provided in the supplemental material 1

^‡^Part of the data used in the preparation of this article were obtained from the Alzheimer’s Disease Neuroimaging Initiative (ADNI) database (adni.loni.usc.edu). The ADNI was launched in 2003 as a public-private partnership, led by Principal Investigator Michael W. Weiner, MD. The primary goal of ADNI has been to test whether serial magnetic resonance imaging, positron emission tomography, other biological markers, and clinical and neuropsychological assessment can be combined to measure the progression of mild cognitive impairment and early Alzheimer’s disease. For up-to-date information, see www.adni-info.org

*Abbreviations*: *yHC* young healthy controls, *eHC* elderly healthy controls, *AD* Alzheimer’s disease dementia, *SCD* subjective cognitive decline, *MCI* mild cognitive impairment, *SUVR* standardized uptake value ratio, *PET* positron emission tomography, *M* male, *F* female, *Aβ* amyloid-beta, *ROC* receiver operating characteristic

### Image analysis

#### ^18^F-Florbetaben acquisition and image processing

Details on the PET image acquisition and reconstruction are provided in the respective original publication of the studies used (Table 1). In short, all subjects underwent a 20-min PET scan (4 × 5 min dynamic frames) starting at least 90 min after intravenous injection of 300 MBq ± 20% of ^18^F-florbetaben followed by a 10-mL saline flush. PET scans were reconstructed using Ordered Subsets Expectation Maximization (OSEM) algorithm using 4 iterations and 16 subsets (zoom = 2) or comparable reconstruction. Corrections were applied for attenuation, scatter, randoms, and dead time. Three-dimensional volumetric T1-weighted brain magnetic resonance imaging (MRI) data was also collected. Then, a Gaussian smoothing kernel was applied to all the scans to bring the ^18^F-florbetaben PET images from different scanner models to a uniform 8 × 8 × 8 mm spatial resolution. The Gaussian smoothing kernel for each scanner was determined using previously acquired Hoffman brain phantoms [27]. Image analysis of ^18^F-florbetaben PET scans was conducted using SPM8 (https://www.fil.ion.ucl.ac.uk/spm/software/spm8/). Motion correction was performed on each PET frame, and an average PET image was generated. Then, the average PET scan was co-registered to its associated T1-weighted MRI scan. Subsequently, the MRI image was segmented into gray matter, white matter, and cerebrospinal fluid, and spatially normalized to the standard MNI (Montreal Neurological Institute) space. The normalization transformation was applied to the co-registered PET scans and gray matter probability maps.

##### MRI-derived ROIs

Regions of interest (ROIs) were defined as the intersection between the standard Automated Anatomic Labeling (AAL) atlas [28] and the normalized gray matter segmentation map thresholded at a probability level of 0.2. ROIs included the cerebellar gray matter and frontal (orbitofrontal and prefrontal), lateral temporal (inferior and superior), occipital, parietal, precuneus, anterior cingulate, posterior cingulate, striatum, amygdala, and thalamus. Mean radioactivity values were obtained from each ROI without correction for partial volume effects applied to the PET data. SUVR was calculated as the ratio of the activity in the target ROI to the activity in the reference region ROI (cerebellar gray matter). A composite SUVR was calculated by unweighted averaging the SUVR of the 6 cortical regions (frontal, lateral temporal, occipital, parietal, anterior, and posterior cingulate cortices) [29].

##### Calibration to centiloid (CL) scale

Given that SUVR values may depend on the tracer used and analytical methods, all the analysis of this paper were provided in CL scale to make the cutoffs useful to other groups or when using other amyloid tracers. Centiloid (CL) values were calculated for each ^18^F-florbetaben PET using the method described by Klunk et al. [30]. ROIs downloaded from the Global Alzheimer’s Association Interactive Network (GAAIN) website (http://www.gaain.org) for the cerebral cortex and the whole cerebellum were applied to the normalized ^18^F-florbetaben PET. Cortical SUVR was calculated as the ratio of the activity in the cortex to the activity in the reference region ROI (whole cerebellum). Finally, the CL values were calculated (CL = 153.4 ⋅ SUVR − 154.9) [31]. The in-house implementation of the standard CL analysis was validated using data freely accessible at the GAAIN website (http://www.gaain.org). SUVRs and CL values from the validation dataset were compared by means of linear correlation to those reported by Klunk et al. [30] (SUVR_Klunk_, CL_Klunk_). The in-house implementation of standard CL analysis passed all the validation criteria described by Klunk et al. [30] being SUVR = 1.01 SUVR_Klunk_ − 0.01 (*R*^2^ = 0.998) and CL = 1.00 CL_Klunk_ + 0.00 (*R*^2^ = 1.00) the regression lines when the whole cerebellum was used as the reference region.

#### ^18^F-Flortaucipir (^18^F-AV1451) acquisition and image processing

Details on the PET image acquisition and reconstruction are provided in ADNI3 PET technical procedures manual (https://adni.loni.usc.edu/wp-content/uploads/2012/10/ADNI3_PET-Tech-Manual_V2.0_20161206.pdf). In short, all subjects underwent a 30-min PET scan (6 × 5 min dynamic frames) starting at 75 min after intravenous injection of 370 MBq ± 10% of flortaucipir. Image analysis of ^18^F-flortaucipir PET scans was performed using the same methods described for ^18^F-florbetaben PET analysis. Cortical ROIs extracted from the AAL atlas included the mesial temporal (average of amygdala, hippocampus, and parahippocampus), fusiform gyrus, inferior lateral temporal, parietal cortices, and cerebellar gray matter. SUVR was calculated as the ratio of the activity in the cortical ROIs to the activity in the reference region (cerebellar gray matter excluding vermis and anterior lobe cerebellar surrounding the vermis).

### Visual assessment

Amyloid PET scans from a subset of 416 participants (*n* = 65 (yHC), *n* = 66 (eHC), *n* = 168 (SCD), *n* = 44 (MCI), *n* = 73 (AD)) (datasets #1, #2, #3, and #4) were visually assessed by independent blinded readers using the method described in Seibyl et al. [10]. The readers were blinded to any structural information (CT or MRI) and different for each of the studies included in the manuscript. The subjects used to generate cutoffs for the detection of established Aβ amyloid pathology (dataset #2) and MCI subjects (dataset #4) were read by 3 independent blinded readers with previous experience reading FBB scans and the final assessment was based on the majority read (i.e., agreement of the majority of readers).

### SUVR cutoff development and definition of the gray zone

#### Development of an SUVR cutoff for the detection of early Aβ deposition (SUVR_early_)

A group of visually Aβ-negative cognitively normal yHC (dataset #1) were used to develop an SUVR cutoff to detect early amyloid deposition. A Shapiro-Wilk test was applied to ascertain that the distribution of each regional SUVR was not significantly different from the Gaussian distribution. Then, the regional SUVR_early_ cutoffs were calculated as 2 standard deviations above the mean SUVR of the yHC.

#### Development of an SUVR cutoff for the detection of established Aβ pathology (SUVR_estab_)

The established pathology SUVR cutoff was derived using receiver operating characteristic (ROC) analysis to ascertain the optimal threshold for the sensitivity and specificity calculation on a sample of visually Aβ-negative eHC and visually Aβ-positive AD dementia patients (dataset #2) [13]. The SUVR that provided the highest Youden’s index (sensitivity + specificity − 1) was selected. In cases that several SUVR provided the same Youden’s index, the SUVR with higher specificity was selected. Global visual assessment as described by Seibyl et al. was used as the standard of truth [10].

#### Definition of the “gray zone”

Given the developed SUVR cutoffs, three groups were defined within the SUVR continuum: Aβ-negative subjects (SUVR < SUVR_early_), early Aβ deposition or “gray zone” (SUVR_early_ ≤ SUVR ≤ SUVR_estab_), and Aβ-positive subjects with established amyloid pathology (SUVR_estab_ < SUVR).

### Characterization of earliest in vivo signal and SUVR cutoff assessment

#### Characterization of earliest in vivo signal in amyloid PET images and topographical distribution

Given that each brain region may have different non-specific binding and dynamic SUVR ranges, direct comparison of SUVR across regions cannot be used to extract the regions showing the earliest amyloid deposition. The assessment of early amyloid deposition assumed that amyloid accumulation follows a logistic growth [32]:

$$ \mathrm{SUVR}(t)=\mathrm{NS}+\frac{K}{1+{e}^{-r\Big(t-{T}_{50}}\Big)} $$

where *t* is the time through the accumulation process, SUVR(*t*) is the regional SUVR at time *t*, NS is the tracer non-specific binding, *r* is the exponential uninhibited growth rate, *K* is the carrying capacity, and *T*_50_ is the time of half-maximal Aβ carrying capacity. NS, *K*, *r*, and *T*_50_ could be different for each region. However, the logistic growth model could not be fitted given the cross-sectional nature of the data used in this work (i.e., individual times through the accumulation process are unknown). Instead, half of the maximum amyloid carrying capacity (SUVR(*t* = *T*_50_)) reached when *t* = *T*_50_ was used to identify those regions that show earliest amyloid signal using PET. In this study, it was hypothesized that *T*_50_ will be smallest in regions with early amyloid deposition.

$$ \mathrm{SUVR}\left(t={T}_{50}\right)=\mathrm{NS}+\frac{K}{2} $$

where NS was estimated from the regional mean SUVR of the visually Aβ-negative yHCs (NS=SUVR_yHC_) and *K* was estimated from the difference between the regional mean of visually Aβ-positive AD dementia subjects (SUVR_AD_) and SUVR_yHC_ (*K* = SUVR_AD_ − SUVR_yHC_).

$$ \mathrm{SUVR}\left(t={T}_{50}\right)=\frac{{\mathrm{SUVR}}_{\mathrm{AD}}+{\mathrm{SUVR}}_{\mathrm{yHC}}}{2} $$

Then, a regional ΔSUVR was derived to characterize the location of a subject in the AD continuum as follows: ΔSUVR = SUVR − SUVR(*t* = *T*_50_). The ΔSUVR takes positive values in those subjects and regions that are above SUVR(*t* = *T*_50_) and close to the SUVR of subjects with AD dementia and negative values in those subjects and regions that are close to SUVR of yHC. ΔSUVR score was compared across regions. Those regions that reached half of the maximum amyloid carrying capacity (ΔSUVR = 0) earlier were considered the regions that show earliest amyloid deposition. Amygdala, thalamus, and striatum, which have a limited dynamic SUVR range between yHC and subjects with AD dementia due to low tracer accumulation, were not included in the interpretation of ΔSUVRs.

#### Assessment of Aβ accumulation

In this study, it was hypothesized that subjects with SUVR in the “gray zone” are in the initial stages of Aβ accumulation. To test this hypothesis, Aβ accumulation was assessed in two samples of SCD and MCI subjects with longitudinal ^18^F-florbetaben PET scans (datasets #3 and #4). To estimate the annual SUVR increase, a linear regression model was fitted to each subject’s data, SUVR = *α* ⋅ *t* + *β*, where *α* and *β* are the coefficients of the model, and *t* is the scan time in years. The annual SUVR increase was obtained from *α*. The percent of Aβ deposition per year (Aβ_dep_) was determined as Aβ_dep_ = 100 ⋅ *α*/SUVR_B_ where SUVR_B_ is the SUVR at baseline. Subsequently, the average annual SUVR increase (*α*) in each sample was tested statistically by means of a *t*-test to demonstrate that those subjects in the “gray zone” are in the process of accumulating Aβ (i.e., (*H*_0_ : *α* = 0; *H*_1_ : *α* > 0). Likewise, annual CL increase (*α*_CL_) was estimated using a linear regression model fitted to each subject’s data, CL = *α*_CL_ *t* + *β*_CL_, where *α*_CL_ and *β*_CL_ are the coefficients of the model and *t* is the scan time in years.

#### Assessment of tau deposition

In subjects that underwent a tau PET scan (dataset #5), ^18^F-flortaucipir SUVR (mean ± SD) was estimated in the three cutoff-based groups (Aβ-negative subjects, subjects in the “gray zone”, and Aβ-positive subjects with established amyloid pathology) and compared by means of a *t*-test.

#### Sensitivity of visual assessment to detect early amyloid accumulation

In those subjects assessed visually (datasets #1, #2, #3, and #4), the proportion of visually Aβ-positive scans was estimated in the three cutoff-based groups (Aβ-negative subjects, subjects in the “gray zone”, and Aβ-positive subjects with established amyloid pathology) to assess the sensitivity of visual assessment to detect early amyloid accumulation.

## Results

### Development of SUVR_early_ cutoff

**Fig. 1:**
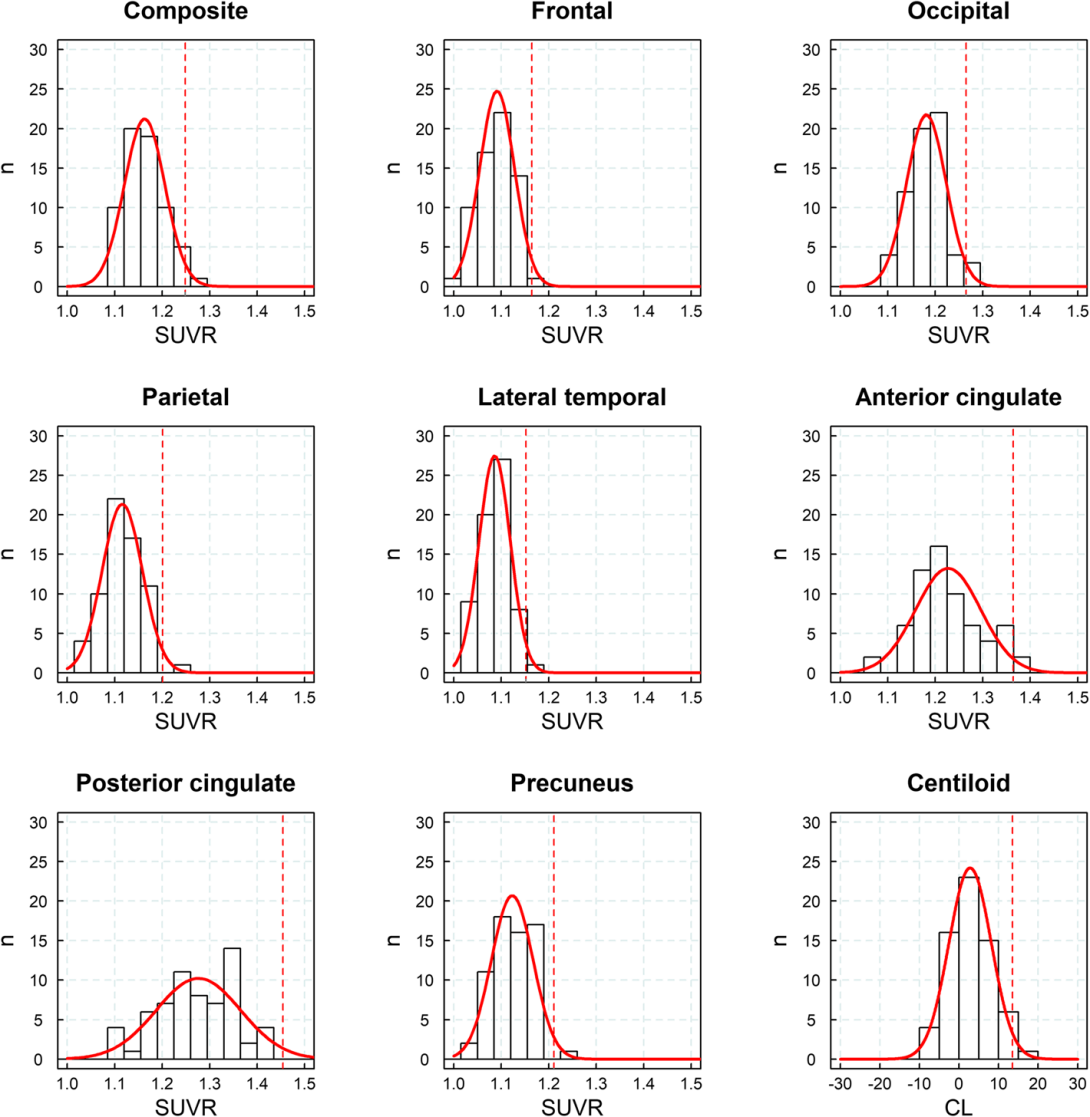
Histograms of standardized uptake value ratios (SUVRs) and cortex centiloids (CLs) in young healthy controls (*n* = 65, dataset #1), fitted Gaussian distribution (red), and SUVR cutoff derived for the detection of early Aβ pathology (red dashed line)

A sample of cognitively normal yHC was used to develop the SUVR cutoff to detect early amyloid deposition. The distribution of SUVRs in yHC did not statistically differ from a Gaussian distribution being the Shapiro-Wilk test non-significant (*p* > 0.05) in any of the regions analyzed (Fig. 1, Table 2). The composite SUVR using MRI-derived ROIs in the yHC was 1.16 ± 0.04 (mean ± SD) resulting in a SUVR_early_ cutoff of 1.25 (Fig. 1, Table 2). The determined SUVR_early_ cutoff differed across regions ranging from 1.15 (lateral temporal cortex) and 1.45 (posterior cingulate cortex) (Table 2). When the standard CL ROIs were applied, the mean of the yHC was 1.03 ± 0.03 (2.82 ± 5.36 CL) and the resulting cutoff (CL_early_) was 1.10 (13.5 CL) (Table 2).

**Table 2:** SUVRs of yHC (dataset #1, *n* = 65) and cutoffs for the detection of early Aβ accumulation (between parenthesis)

Method	Region	SUVR_yHC_ (cutoff)	*p*
MRI-derived ROIs	Frontal	1.09 ± 0.04 (1.16)	0.83
	Lateral temporal	1.09 ± 0.03 (1.15)	0.41
	Occipital	1.18 ± 0.04 (1.26)	0.93
	Parietal	1.12 ± 0.04 (1.20)	0.92
	Anterior cingulate	1.23 ± 0.07 (1.36)	0.13
	Posterior cingulate	1.28 ± 0.09 (1.45)	0.43
	Precuneus	1.12 ± 0.04 (1.21)	0.22
	Composite	1.16 ± 0.04 (1.25)	0.25
CL ROIs	Cortex	1.03 ± 0.03 (1.10) 2.82 ± 5.36 CL (13.54 CL)	0.32

*yHC* young healthy controls, *SUVR*_*yHC*_ SUVR (mean ± SD) of the young healthy controls, *Aβ* amyloid-beta, *p p*-values from the Shapiro-Wilk test to assess that SUVR values are normally distributed (*p* < 0.05 = significant differences from the normality)

### Development of the SUVR_estab_ cutoff

**Fig. 2:**
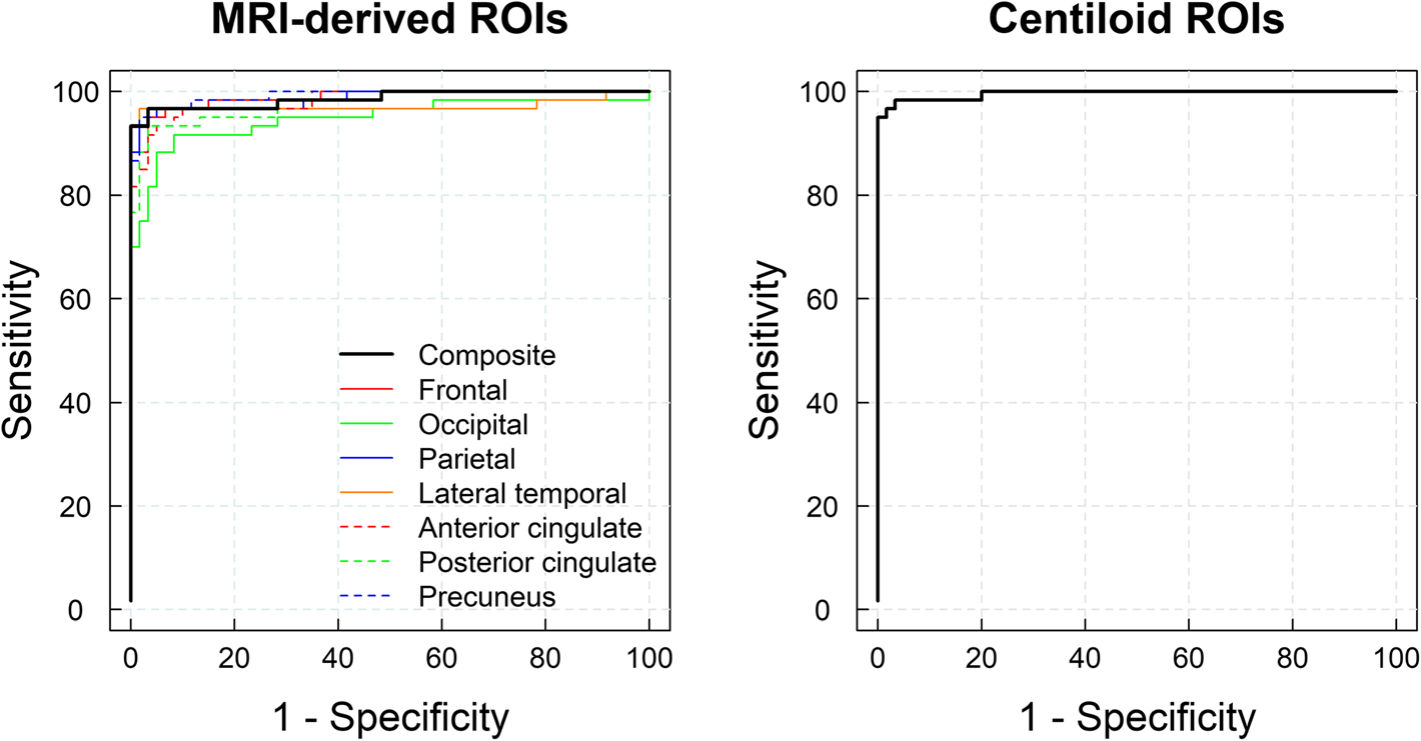
Receiver operating characteristic curves obtained using MRI-derived regions of interest (ROIs, left) and centiloid (right) used to derive standardized uptake value ratio cutoffs for the established Alzheimer’s disease pathology from a group of elderly healthy controls (*n* = 66) and subjects with AD dementia (*n* = 73, dataset #2)

ROC analysis using visual assessment as the standard of truth resulted in SUVR_estab_ cutoffs ranging from 1.26 (lateral temporal and parietal cortices) to 1.47 (posterior cingulate cortex). The SUVR cutoff (MRI-derived ROIs) for the composite region was 1.38 (Fig. 2, Table 3). When the standard CL analysis was applied, the SUVR_estab_ and CL_estab_ cutoff obtained were 1.24 and 35.7 CL, respectively.

**Table 3:** SUVRs of eHC (*n* = 66) and AD subjects (*n* = 73) (dataset #2) and cutoffs for the detection of established Aβ pathology

Method	Region	SUVR_eHC_	SUVR_AD_	SUVR_cutoff_	Sensitivity	Specificity	AUC
MRI-based ROIs	Frontal	1.15 ± 0.07	1.57 ± 0.19	1.31	95%	97%	0.98
	Lateral temporal	1.15 ± 0.05	1.51 ± 0.17	1.26	97%	98%	0.96
	Occipital	1.20 ± 0.06	1.43 ± 0.16	1.29	88%	95%	0.94
	Parietal	1.13 ± 0.08	1.51 ± 0.16	1.26	97%	98%	0.98
	Anterior cingulate	1.28 ± 0.09	1.70 ± 0.22	1.43	92%	97%	0.98
	Posterior cingulate	1.33 ± 0.09	1.77 ± 0.21	1.47	93%	97%	0.97
	Precuneus	1.15 ± 0.08	1.60 ± 0.19	1.28	95%	98%	0.98
	Composite	1.21 ± 0.06	1.58 ± 0.17	1.38	93%	100%	0.98
CL ROIs	Cortex	1.05 ± 0.06 6.8 ± 8.8 CL	1.54 ± 0.22 81.0 ± 33.2 CL	1.24 35.7 CL	95%	100%	0.98

*eHC* elderly healthy controls, *Aβ* amyloid-beta, *SUVR*_*eHC*_ SUVR (mean ± SD) of the elderly healthy controls, *SUVR*_*AD*_ SUVR (mean ± SD) of the Alzheimer’s disease patients, *AUC* area under the receiver operating curve, *SUVR*_*cutoff*_ SUVR cutoff obtained from the ROC analysis using visual assessment as standard of truth, *MRI* magnetic resonance imaging, *ROI* region of interest, *CL* centiloid

### Earliest in vivo signal in amyloid PET images and topographical distribution

**Fig. 3:**
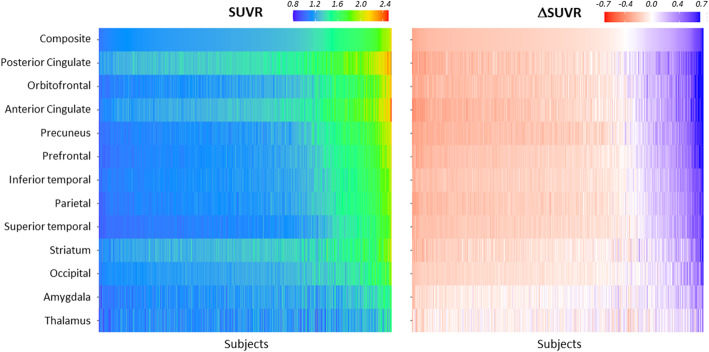
Heat maps of standardized uptake value ratios (SUVRs, left) and ΔSUVRs (=SUVR − SUVR(*t* = *T*_50_)) (right) of all the participants in the analysis (*n* = 686, datasets #1, #2, #3, #4, and #5). Each column of the heat map represents one subject of the sample. The subjects were sorted according to their composite SUVR (increasing from left to right)

Posterior and anterior cingulate cortices followed by precuneus, frontal, and inferior temporal cortices were the regions that showed earlier elevated SUVR values (Fig. 3, left panel). However, given that each region has a different non-specific uptake and SUVR dynamic range, the regional SUVRs were compared against the half maximum amyloid carrying capacity by means of ΔSUVR (ΔSUVR = SUVR − SUVR(*t* = *T*_50_)) to determine the regions that show earliest amyloid accumulation (Fig. 3, right panel). Cingulate cortices (anterior and posterior), precuneus, and orbitofrontal were the regions that first showed pathological Aβ PET tracer retention followed by prefrontal, inferior lateral temporal, parietal, and occipital cortices (Fig. 3, right panel). Other regions that showed tracer retention and differences from yHC were the striatum and the amygdala.

### Aβ deposition in subjects with SCD

**Fig. 4:**
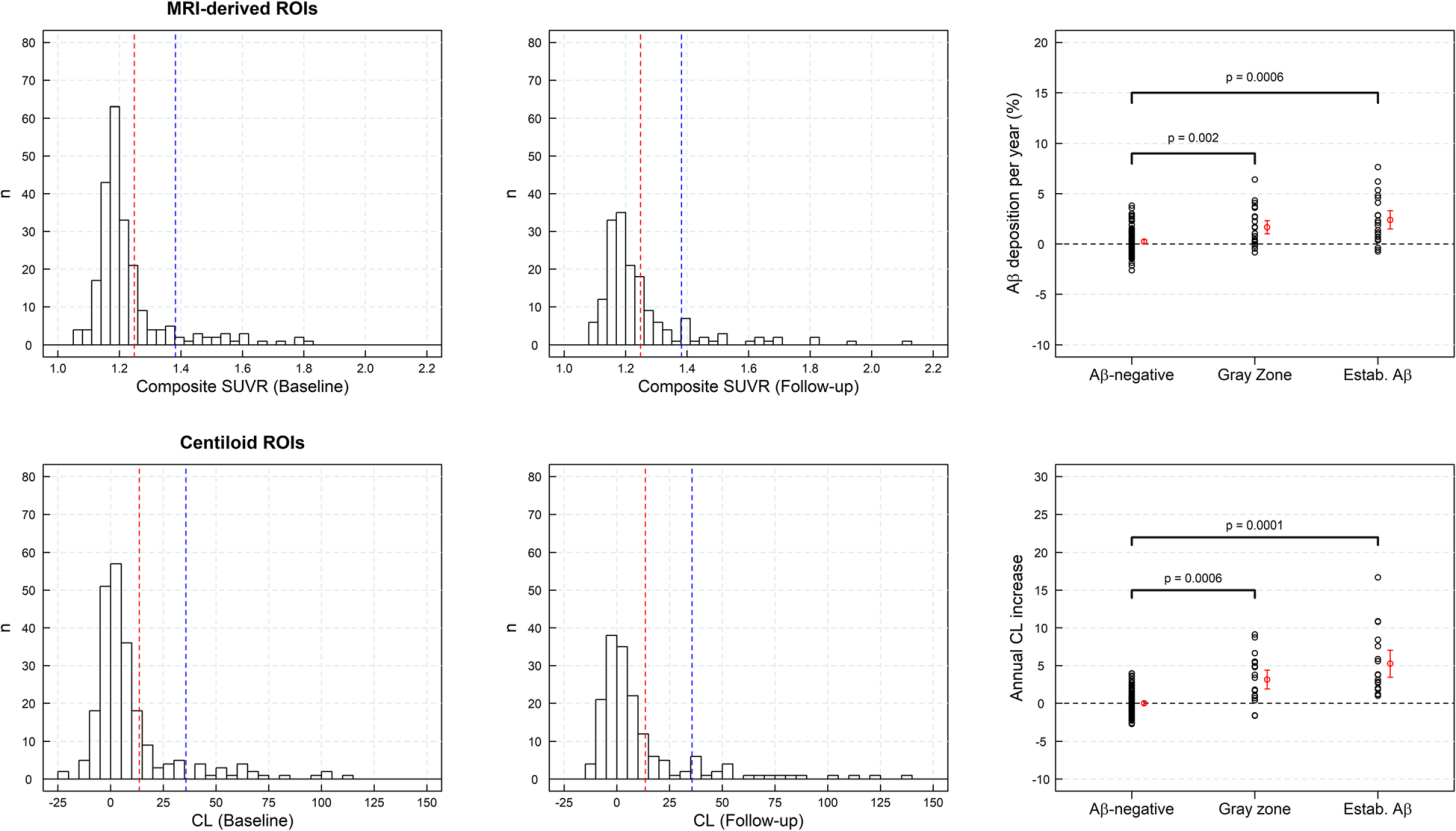
Histograms of composite standardized uptake value ratios (SUVRs) and centiloids (CLs) for the sample of subjective cognitive decline (SCD) (*n* = 168, dataset #3) subjects at baseline (first column) and at follow-up (central column). Red and blue lines represent the SUVR abnormality cutoffs for early Aβ detection and established Aβ pathology, respectively. The rate of Aβ accumulation in SCD (and 95% confidence interval in red) in three categories of the composite SUVR continuum (Aβ-negative, gray zone, and established Aβ deposition) is shown on the right column. ROI region of interest

SUVR histograms derived from a sample of subjects with SCD showed a peak coincident with the Gaussian function fitted to the sample of yHC with a tail with higher SUVRs that increased numbers at follow-up (Fig. 4). The rate of amyloid accumulation increased significantly in those subjects with SUVR in the “gray zone” or with established Aβ deposition in comparison with Aβ-negative subjects (*p* < 0.002) (Fig. 4). The subjects with SUVRs in the “gray zone” and established Aβ deposition had rates of Aβ accumulation statically different from zero (*p* < 0.001) (1.66 ± 1.86%/year (composite) and 2.40 ± 2.37%/year (composite), respectively) (Fig. 4, Table 4). Similar results were obtained when the CL analysis was used (1.81 ± 1.86 CL/year (*p* < 0.001)) (gray zone), 2.38 ± 1.82 CL/year (*p* < 0.001) (established Aβ pathology)). In general, the Aβ accumulation was significantly larger for subjects in the gray zone and established Aβ deposition than in Aβ-negative subjects (Fig. 4, Table 4).

**Table 4:** Regional percent of Aβ deposition per year in a sample of subject with SCD (*n* = 168, dataset #3)

		Percent Aβ deposition per year		
Method	Region	Aβ-negative	Gray zone	Established Aβ pathology
MRI-based ROIs	Frontal	− 0.01 ± 1.15 (*p* = 0.52)	1.08 ± 1.91 (*p* < 10^−3^)	2.72 ± 2.53 (*p* < 10^−4^)
	Lateral temporal	0.08 ± 0.99 (*p* = 0.21)	1.07 ± 1.61 (*p* < 10^−4^)	2.05 ± 2.18 (*p* < 10^−3^)
	Occipital	0.36 ± 1.13 (*p* < 10^−3^)	0.50 ± 1.74 (*p* = 0.38)	1.85 ± 2.18 (*p* < 10^−3^)
	Parietal	0.12 ± 1.29 (*p* = 0.16)	1.39 ± 2.10 (*p* = 0.02)	2.61 ± 2.28 (*p* < 10^−5^)
	Anterior cingulate	0.17 ± 1.81 (*p* = 0.13)	1.42 ± 2.12 (*p* = 0.10)	2.37 ± 3.07 (*p* = 0.001)
	Posterior cingulate	0.71 ± 1.72 (*p* < 10^−5^)	N.A	3.14 ± 2.46 (*p* < 10^−7^)
	Precuneus	0.31 ± 1.37 (*p* = 0.006)	N.A	N.A
	Composite	0.24 ± 1.24 (*p* = 0.02)	1.66 ± 1.86 (*p* < 10^−3^)	2.40 ± 2.37 (*p* < 10^−3^)
CL	Cortex	0.00 ± 0.89 (*p* = 0.53)	1.81 ± 1.86 (*p* < 10^−3^)	2.38 ± 1.82 (*p* < 10^−4^)

*SCD* subjective cognitive decline, *N.A* not available (As Aβ-negative, gray zone, and established Aβ pathology were defined regionally using cutoffs reported in Tables 2 and 3, there were not enough regional standardized uptake values (SUVRs) to calculate percent Aβ deposition per year in some regions), *Aβ* amyloid-beta, *MRI* magnetic resonance imaging, *ROI* region of interest, *CL* centiloid. *p*-values testing whether percent Aβ deposition per year is significantly larger than zero are given in parenthesis

### Progression to AD dementia in MCI subjects

**Fig. 5:**
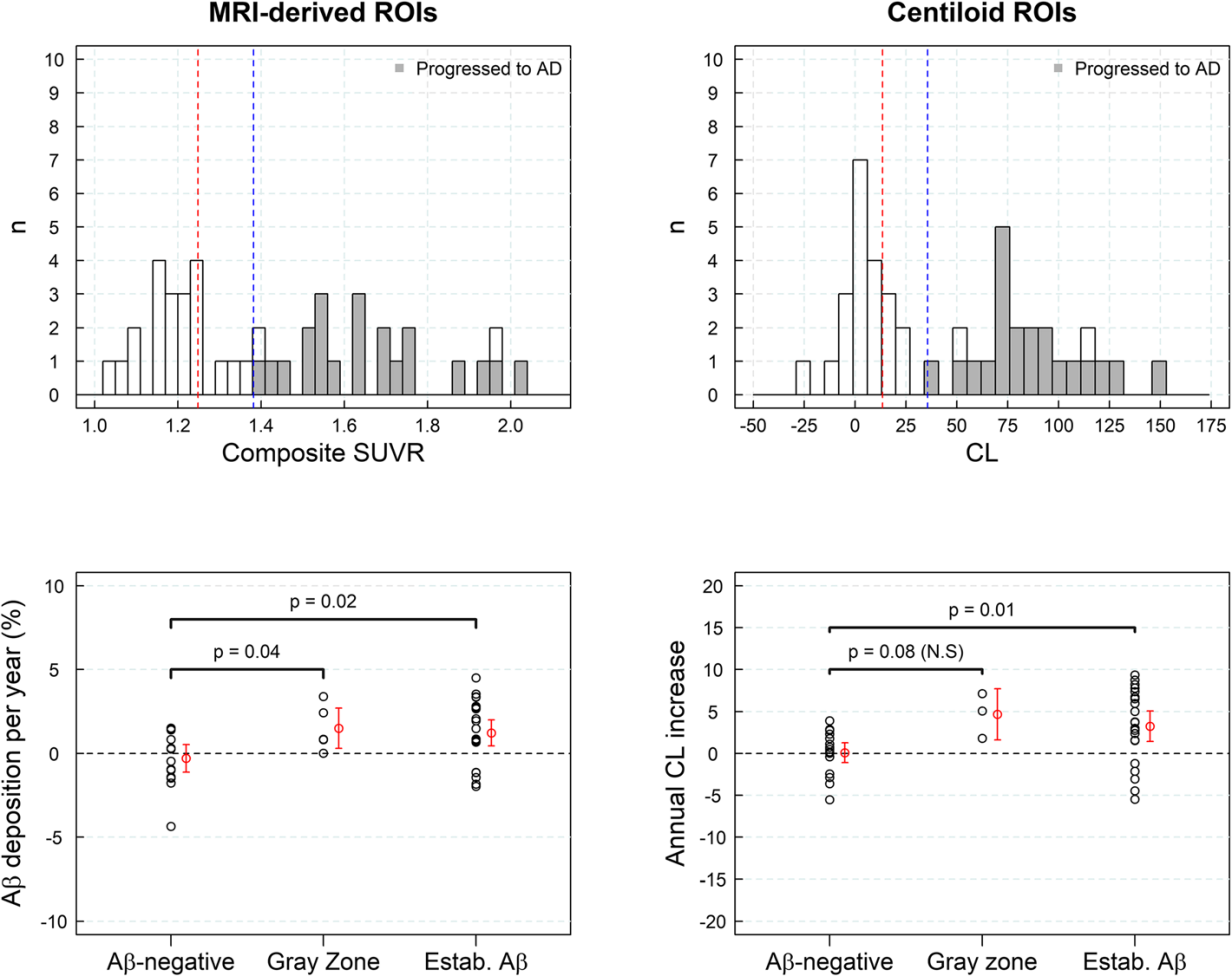
Histograms of composite standardized uptake value ratios (SUVRs) and centiloids (CLs) for the sample of mild cognitive impairment (MCI) (*n* = 44, dataset #4) subjects are shown on the top row. Subjects that progressed to Alzheimer’s disease (AD) dementia after a 4-year clinical follow-up are shown in gray. Red and blue lines represent the SUVR abnormality cutoffs for early Aβ detection and established Aβ pathology, respectively. The rate of Aβ accumulation in MCI subjects (and 95% confidence interval in red) in three categories of the composite SUVR continuum: Aβ-negative, gray zone, and with established Aβ deposition, is shown on the bottom row. ROI region of interest

SUVR histograms obtained from the MCI subjects showed a broad range of SUVRs (Fig. 5). In general, the rate of amyloid accumulation increased significantly in those subjects with SUVR in the “gray zone” or with established Aβ deposition in comparison with Aβ-negative subjects (*p* < 0.05). However, the difference between Aβ-negative subjects and subjects in the “gray zone” did not reach statistical significance when CL ROIs were used (Fig. 5). In the composite region, the rate of Aβ accumulation in the “gray zone” (1.51 ± 1.38%/year (*p* = 0.04) and for “established Aβ deposition” (1.23 ± 1.90%/year (*p* = 0.004)) was significantly different from zero (Fig. 5) while no accumulation was found in Aβ-negative subjects (− 0.29 ± 1.68%/year (*p* = 0.74)) (Table 5). None of the Aβ-negative subjects or subjects in the “gray zone” progressed to AD dementia after a 4-year clinical follow-up. Twenty-one subjects (91%) with SUVR above SUVR_estab_ progressed to AD dementia after 4 years (Fig. 5).

**Table 5:** Regional percent of Aβ deposition per year in a sample of MCI subjects (*n* = 44, dataset #4)

		Percent Aβ deposition per year		
Method	Region	Aβ-negative	Gray zone	Established Aβ pathology
MRI-based ROIs	Frontal	− 0.49 ± 2.52 (*p* = 0.73)	0.85 ± 2.20 (*p* = 0.19)	1.37 ± 2.02 (*p* = 0.002)
	Lateral temporal	0.15 ± 1.83 (*p* = 0.40)	0.88 ± 1.58 (*p* = 0.08)	1.66 ± 1.90 (*p* < 10^−3^)
	Occipital	0.30 ± 1.64 (*p* = 0.21)	0.24 ± 1.78 (*p* = 0.44)	0.94 ± 2.28 (*p* = 0.055)
	Parietal	− 0.48 ± 1.72 (*p* = 0.87)	1.39 ± 1.23 (*p* = 0.09)	1.12 ± 1.88 (*p* = 0.009)
	Anterior cingulate	− 0.79 ± 2.49 (*p* = 0.87)	0.97 ± 0.22 (*p* = 0.01)	0.76 ± 2.51 (*p* = 0.08)
	Posterior cingulate	0.71 ± 1.41 (*p* = 0.04)	N.A	1.58 ± 2.26 (*p* = 0.001)
	Precuneus	0.01 ± 1.37 (*p* = 0.49)	1.24 ± 1.56 (*p* = 0.11)	1.44 ± 2.31 (*p* = 0.004)
	Composite	− 0.29 ± 1.68 (*p* = 0.74)	1.51 ± 1.38 (*p* = 0.04)	1.23 ± 1.90 (*p* = 0.004)
CL	Cortex	0.08 ± 1.62 (*p* = 0.43)	2.62 ± 1.47 (*p* = 0.045)	1.41 ± 1.82 (*p* = 0.001)

*MCI* mild cognitive impairment, *N.A* not available (As Aβ-negative, gray zone, and established Aβ pathology were defined regionally using cutoffs reported in Tables 2 and 3, there were not enough regional standardized uptake values (SUVRs) to calculate percent Aβ deposition per year in some regions), *Aβ* amyloid-beta, *MRI* magnetic resonance imaging, *ROI* region of interest, *CL* centiloid. *p*-values testing whether percent Aβ deposition per year is significantly larger than zero are given in parenthesis

### Association between Aβ load and tau deposition

**Fig. 6:**
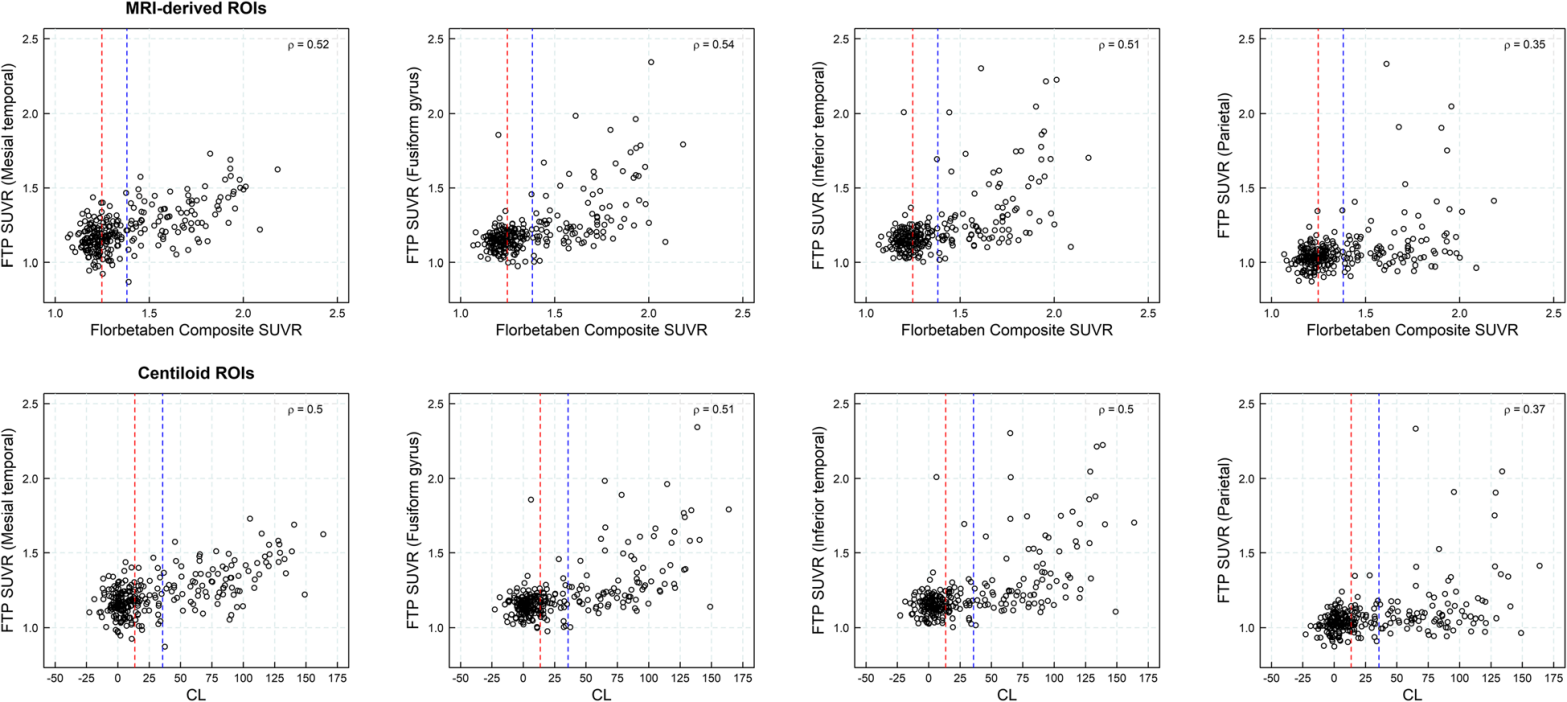
Scatter plots of Flortaucipir (FTP) standardized uptake value ratios (SUVRs) versus ^18^F-florbetaben composite SUVRs using MRI-based regions of interest (ROIs, top row) and FTP SUVRs versus centiloids (CLs, bottom row) (*n* = 270, dataset #5). Red and blue lines represent the composite SUVR abnormality cutoffs for early Aβ detection and established Aβ pathology, respectively

Figure 6 shows the association between amyloid load measured with ^18^F-florbetaben and tau load measured with [^18^F] flortaucipir (*ρ* = 0.35 (parietal)–0.54 (fusiform gyrus) (*ρ*: Spearman correlation coefficient); *p* < 0.0001) (Table 6, Fig. 6). Tau deposition was rarely observed in Aβ-negative subjects and subjects in the gray zone: (SUVR(^18^F-flortaucipir) =1.15 ± 0.08 (Aβ-negative), 1.16 ± 0.09 (gray zone) (Fusiform gyrus)), but increased in subjects with established Aβ pathology (SUVR(^18^F-flortaucipir) = 1.35 ± 0.24 (Fusiform gyrus)) (Table 6).

**Table 6:** Regional ^18^F-flortaucipir SUVRs by amyloid group (*n* = 270, dataset #5)

		^18^F-Flortaucipir SUVR		
Method	Region	Aβ-negative	Gray zone	Established Aβ pathology
MRI-based ROI	Mesial temporal	1.16 ± 0.09	1.18 ± 0.10 (*p* = 0.51)	1.32 ± 0.15 (*p* < 10^−5^)
	Fusiform gyrus	1.15 ± 0.09	1.16 ± 0.08 (*p* = 0.91)	1.34 ± 0.24 (*p* < 10^−5^)
	Inferior temporal	1.15 ± 0.10	1.17 ± 0.10 (*p* = 0.87)	1.32 ± 0.15 (*p* < 10^−5^)
	Parietal	1.03 ± 0.07	1.05 ± 0.07 (*p* = 0.58)	1.15 ± 0.23 (*p* < 10^−5^)
CL	Mesial temporal	1.16 ± 0.09	1.18 ± 0.11 (*p* = 0.45)	1.33 ± 0.15 (*p* < 10^−5^)
	Fusiform gyrus	1.15 ± 0.08	1.16 ± 0.09 (*p* = 0.88)	1.35 ± 0.24 (*p* < 10^−5^)
	Inferior temporal	1.15 ± 0.05	1.18 ± 0.11(*p* = 0.60)	1.38 ± 0.28 (*p* < 10^−5^)
	Parietal	1.03 ± 0.06	1.06 ± 0.09 (*p* = 0.37)	1.16 ± 0.24 (*p* < 10^−5^)

*SUVR* ^18^F-flortaucipir SUVRs (mean ± SD), *Aβ* amyloid-beta, *MRI* magnetic resonance imaging, *MRI* magnetic resonance imaging, *ROI* region of interest, *CL* centiloid. *p*-values using ANOVA to test whether ^18^F-flortaucipir SUVRs in each group are significantly larger than in Aβ-negative subjects are given in parenthesis

### Sensitivity of visual assessment to detect early amyloid accumulation

**Fig. 7:**
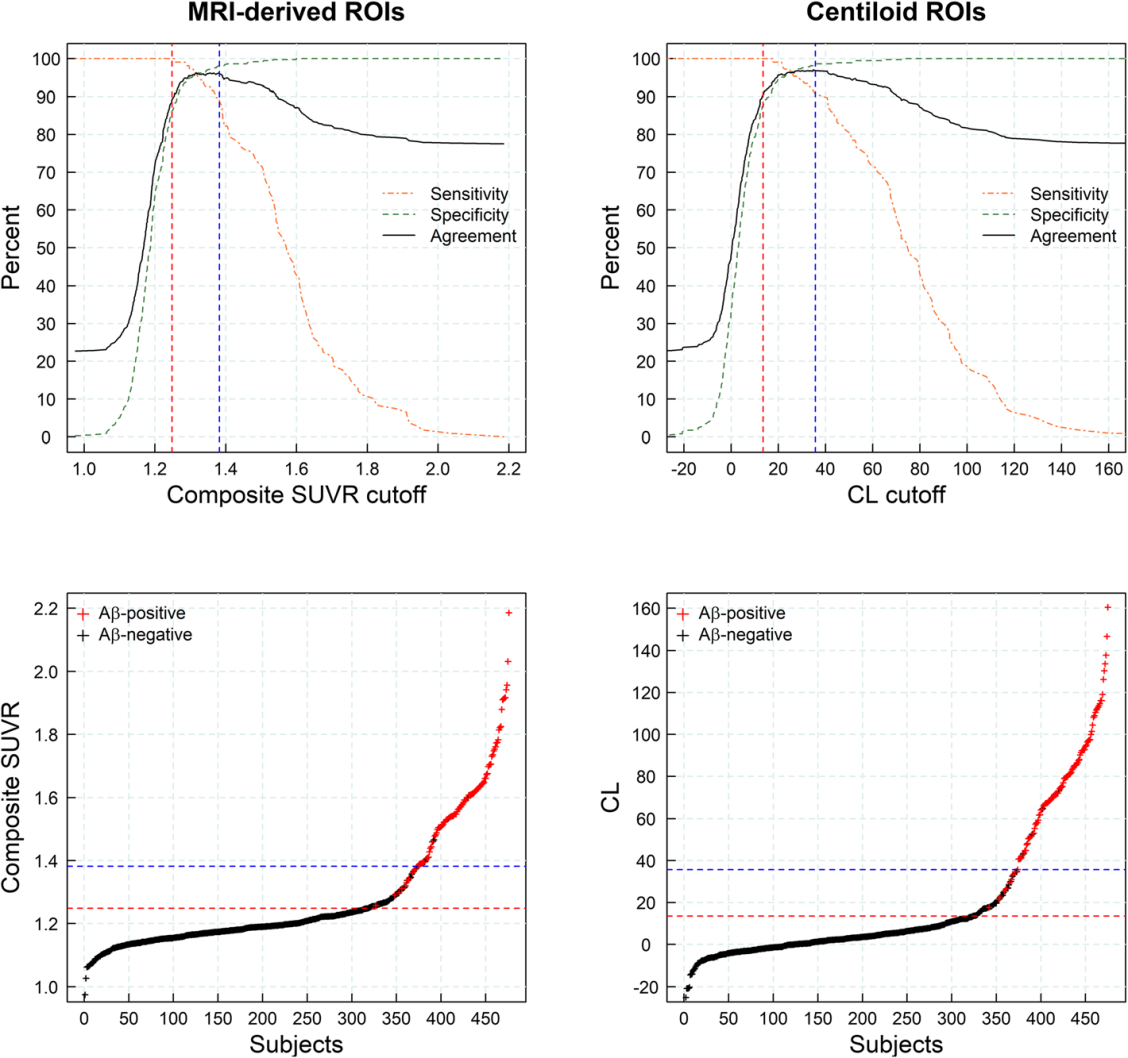
Sensitivities, specificities, and agreement rates between visual assessment and quantitative assessment when using several cutoffs to dichotomize the sample (top row) and composite standardized uptake value ratio (SUVR) versus subject identifier (bottom row) (*n* = 416) (datasets #1, #2, #3, and #4). Red and blue lines represent the composite SUVR abnormality cutoffs for early Aβ detection and established Aβ pathology, respectively. ROI region of interest, CL centiloid

Most of the subjects with established Aβ pathology defined either by SUVR (MRI-derived ROIs) or CL cutoffs were visually assessed as positive (93% and 95%, respectively), while all the subjects in the Aβ-negative group were visually assessed as negative (100%). In the gray zone, only 21.4% (MRI-derived ROIs) and 19.6% (CL) of the subjects were visually assessed as positive. The maximum agreement between visual assessment and quantitative assessment was found for SUVR and CL cutoffs in the upper range of the gray zone, while the agreement decreased in the lower range of the gray zone (Fig. 7).

## Discussion

Currently, observational and interventional studies focus on earlier stages of Aβ deposition, where established SUVR cutoffs to discriminate AD dementia subjects from elderly HC are of limited value. In this study, regional and global quantitative SUVR cutoffs were developed for the detection of early amyloid accumulation and established Aβ pathology using ^18^F-florbetaben PET. A gray zone was defined as the range of SUVR values in subjects having higher SUVR than yHC and less than the values previously used to define visual positivity in patients with AD dementia. The existence of a “gray zone” that may precede visual positivity and the feasibility of identifying subjects in the gray zone using ^18^F-florbetaben PET were corroborated using two quantitative methods (MRI-derived ROIs and CL ROIs). The population in the “gray zone” represents early stages of Aβ deposition characterized by accelerated Aβ accumulation and pre-AD dementia levels of Aβ burden that may precede the alteration of other biomarkers such as tau deposition or clinical symptoms. Although assessment of tau deposition using Flortaucipir PET in mesial temporal structures could be biased due to adjacent choroid plexus uptake, the association between Aβ and tau was strong also in other regions assessed such as fusiform gyrus and inferior lateral temporal cortex. While the agreement between visual and quantitative assessments was excellent for Aβ-negative subjects and subjects with established Aβ pathology, the agreement was modest in the “gray zone.” In these challenging cases, the use of quantitation may help to detect subtle amyloid accumulation. The appropriate definition of a “gray zone” can improve the detection of emerging Aβ pathology in observational, prevention, and therapeutic trials and is key for the screening of asymptomatic population in clinical trials and detection of subjects that will likely accumulate amyloid. Subjects having amyloid values in the gray zone may be the most likely to respond to pharmacological or non-pharmacological interventions because they have early evidence of disease without the cognitive deficits and neuronal loss that signifies AD.

This study is in agreement with a number of recent reports across different tracers converging to the utility of using two cutoffs for amyloid PET abnormality, an early cutoff around CL = 11–17 where pathology may be emerging, and a second around CL = 29–36 where amyloid burden levels correspond to moderate and frequent neuritic plaques (CERAD stages B–C, [33]) by neuropathology. Early cutoffs of 11, 14, and 17 CL have been reported for the FACEHBI, ALFA+, and AMYPAD Prognostic and Natural History Study studies using Gaussian mixture models [34]. Similarly, Salvadó et al. identified two cutoffs based on a direct comparison with established CSF Aβ42 thresholds: CL = 12 to rule out-amyloid pathology and CL = 29 to denote established pathology [35]. Mormino et al. also showed the biological relevance of slight ^11^C-PIB elevations in elderly normal control subjects and provided an estimate for the cutoffs defining the “gray zone” using distribution volume ratios [36]. Finally, La Joie et al. and Doré et al. reported using histopathological confirmation gray zones from 12.2–24.4 and 19–28 CLs, respectively [17, 37].

This study also showed that topographical information can help identify increased signal earlier than traditional global cutoffs, with cingulate cortices (anterior and posterior), precuneus, and orbitofrontal cortices being the first regions to show pathological tracer retention, followed by prefrontal, inferior lateral temporal parietal, and occipital cortices. These results agree with previous publications using PET where precuneus, cingulate, and frontal cortices displayed higher PET signal earlier than the remaining neocortical regions [38–40]. However, recent publications suggest that other regions such as the banks of the superior temporal, not analyzed in this article, may also show early Aβ deposition and subjects with high Aβ in these regions are at increased risk of cognitive decline [41]. Even though regions with “early” amyloid have been identified in this work, these early elevations are subtle, occasionally may not be detectable at the individual level and the amyloid PET signal is highly correlated across all regions. These subtle differences across regions are consistent with some articles reporting that a sigmoidal model fitting amyloid deposition with the same *T*_50_ across brain regions is optimal [32]. In addition, amyloid PET is affected by several technical factors such as the type of camera used, reconstruction methods, corrections applied (e.g., partial volume effect), and quantitative methods used that may have an impact on the regional SUVR estimates. For this reason, topographically defined distribution and early Aβ accumulation measured by PET may not necessarily agree with histopathology findings. Despite these discrepancies with neuropathology results, several studies have shown the utility of amyloid PET topographical quantification in staging AD [21–23], determining the risk of subsequent cognitive decline [23, 25], optimal subject selection for anti-amyloid interventional trials [22, 42], and reducing sample size in anti-amyloid interventional trials [43, 44]. Pascoal et al. also showed that the topographical pattern of individuals with MCI that progress to dementia is “traditionally AD-like,” while that of non-converters includes more temporal and occipital regions instead [24]. In this regard, even though CL ROIs and composite SUVR from MRI-derived ROIs provided overall similar results when determining subject in the “gray zone,” the use of CL and composite SUVR is limited in determining the topographical distribution of Aβ load.

As a limitation of this study, it should be mentioned that SUVR cutoff for the detection of established amyloid pathology was derived using visual assessment as a standard of truth and this may bias the proportion of visually positive scans per group. To clarify this potential bias, a Gaussian mixture model was fitted to the whole population of the study (datasets #1, #2, #3, #4, and #5) confirming the cutoffs previously reported in the manuscript (14 and 32 CL), proportion of positive scans per group and accurate definition of the “gray zone” (supplemental material 2). A second limitation is that SUVR may be biased as a surrogate marker of Aβ load by changes in cerebral blood flow (CBF) or radiotracer clearance [45] and SUVR cutoffs may depend on methodological aspects such as equipment, reconstruction, imaging window, image processing, smoothing, and corrections applied. To minimize this methodological limitation, a harmonization procedure was applied to convert all the images into a common resolution as described in Joshi et al. [27]. Even so, the application of cutoffs developed here should be applied with caution to studies using different methods or non-harmonized data.

## Conclusions

This study supports the utility of two cutoffs for ^18^F-florbetaben amyloid PET abnormality defining a “gray zone”: a first cutoff of 13.5 CL that indicated emerging Aβ pathology and a second cutoff of 35.7 CL where amyloid burden levels correspond to established AD neuropathology findings. These cutoffs define a subset of subjects characterized by pre-AD dementia levels of amyloid burden that may precede the alteration of other biomarkers such as tau deposition or clinical symptoms and accelerated amyloid accumulation. Amyloid PET images in the “gray zone” are more likely to be ambiguous by the current binary global visual assessment methodology. At the MCI stage, the determination of different amyloid loads, particularly low amyloid levels, is useful in determining who will eventually progress to dementia. Quantitation of amyloid provides a sensitive measure in these low-load cases and may help to identify a group of subjects most likely to benefit from intervention.

## Supplementary Information

**Additional file 1.**

**Additional file 2.**

## Data Availability

The datasets generated and/or analyzed during the current study are not publicly available.
