# Supplementary material for: Early detection of amyloid load using 18F-florbetaben PET
Source: Alzheimers Res Ther. 2021 Mar 27;13:67. doi: 10.1186/s13195-021-00807-6 (PMC8005243; doi:10.1186/s13195-021-00807-6)
Supplement: Supplementary file 1 — Additional file 1. [file 13195_2021_807_MOESM1_ESM.pdf]

**Supplemental material 1**

The study population of young cognitively normal subjects (dataset #1) was enrolled at the Institute for Neurodegenerative Disorders, New Haven, CT. The subjects were required to be between 21 and 40 years, MMSE ≥ 28 and CDR=0 of age for inclusion. This study was conducted in accordance with the Declaration of Helsinki and the Good Clinical Practice guidelines and after approval of the local ethics committees of the 2 participating centers, including the Department of Disability Services in Connecticut. Written informed consent was obtained from all the participants or their legally authorized representative. This study was registered with ClinicalTrials.gov, number NCT00928304. Part of this study has been previously reported by Jennings et al. [14]

All participants underwent a screening visit, including a history and physical examination, clinical assessments, and cognitive screening. All participants underwent a $^{18}$F-Florbetaben PET scan as well as a T1-weighted MRI scan. PET images were acquired with a Siemens Exact HR+ tomograph. For the acquisition of PET images, 300 ± 20% MBq of $^{18}$F-Florbetaben was administered as a single slow intravenous bolus (6 s/ml) in a total volume of up to 10 ml, followed by a 10 ml saline flush. A 20-minute scan (4 x 5-minute dynamic frames) was acquired starting at 100 minutes post-injection. PET data were reconstructed using an Ordered Subsets Expectation Maximization algorithm with 4 iterations and 16 subsets (zoom = 2). Corrections were applied for attenuation, scatter, randoms, and dead time.
