# Supplementary material for: Early detection of amyloid load using 18F-florbetaben PET
Source: Alzheimers Res Ther. 2021 Mar 27;13:67. doi: 10.1186/s13195-021-00807-6 (PMC8005243; doi:10.1186/s13195-021-00807-6)

Supplemental material 2

A gaussian mixture model with 3 distributions was fitted to the whole population (datasets #1, #2, #3, #4 and #5). Red, blue, and black lines correspond to the fitted Gaussian functions and the dashed line corresponds to the sum of the three Gaussian functions. The SUVR cutoff for early detection of amyloid load was determined as 2 standard deviations above the left Gaussian distribution (in red). The SUVR cutoff for established pathology was determined as 2 standard deviations above the middle gaussian distribution (in blue). The obtained SUVR cutoffs were 14 and 32 CLs.

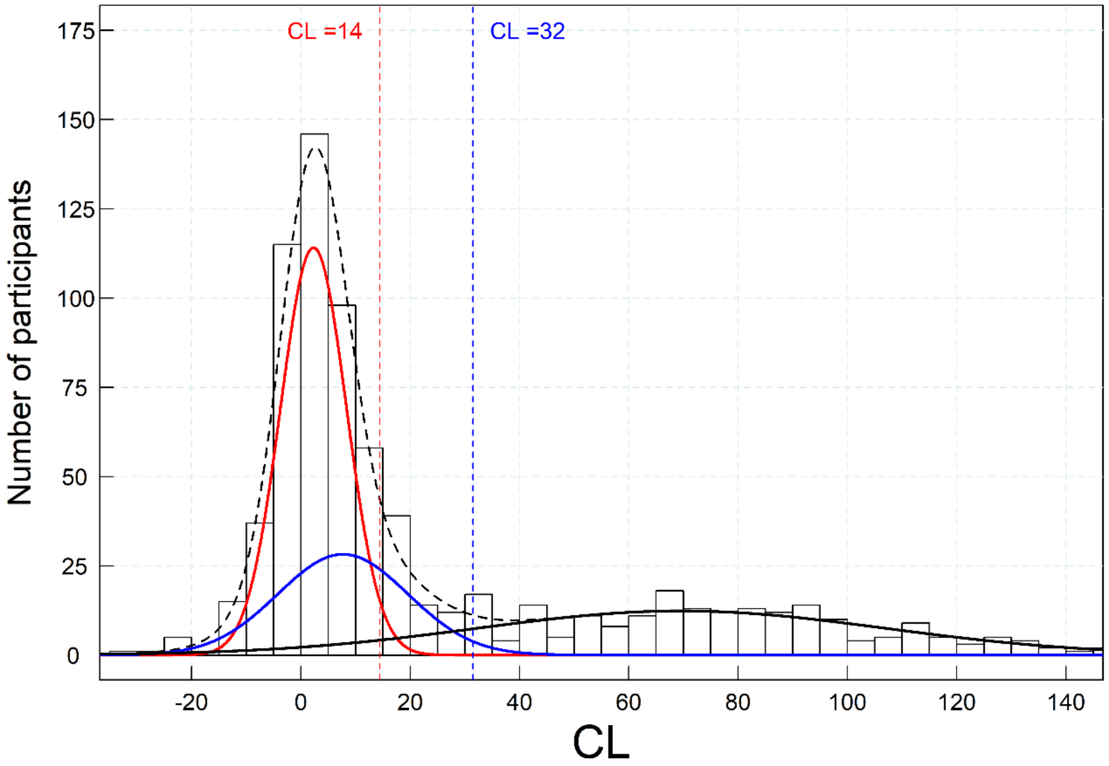

Supplement: Supplementary file 2 — Additional file 2. [file 13195_2021_807_MOESM2_ESM.pdf]
